# Constructing an Adenine Base Editor in *Escherichia coli* for Fine-Tuning the 1,4-Butanediamine Biosynthetic Pathway

**DOI:** 10.3390/microorganisms14081708

**Published:** 2026-08-04

**Authors:** Yanling Sun, Di Ma, Kexin Zhao, Shihao Liu, Weichao Chen, Bixin He, Wei Huang, Fuping Lu, Ming Li

**Affiliations:** 1 State Key Laboratory of Bio-Based Fiber Materials, Tianjin University of Science and Technology, Tianjin 300457, China; 13686327051@163.com (Y.S.); dm13835707932@163.com (D.M.); zkxc0327@163.com (K.Z.); a1270263637@outlook.com (S.L.); m15211434028@163.com (W.C.); 19933611850@163.com (B.H.); 17364456085@163.com (W.H.); 2 Key Laboratory of Industrial Fermentation Microbiology, Ministry of Education, Tianjin University of Science and Technology, Tianjin 300457, China; 3 College of Biotechnology, Tianjin University of Science and Technology, Tianjin 300457, China

**Keywords:** 1,4-butanediamine, base editor, SpRY, promoter engineering, metabolic flux optimization

## Abstract

Adenine base editors (ABEs) can be used to fine-tune metabolic flux by editing gene promoters. However, their targeting scope is constrained by the PAM requirement of Cas9, making it difficult to target promoter regions where NGG motifs are rare. To overcome this limitation, we fused the SpRY variant, a near-PAMless Cas9, with the highly active adenine deaminase TadA8e to construct a near-PAMless ABE. After multi-round optimization, the editor achieved A → G edit at the T7 promoter, along with a bystander C → A edit at neighboring position. The ABE was used to edit the T7 promoter in the 1,4-butanediamine-producing strain, thereby altering the promoter strength and reallocating metabolic flux into 1,4-butanediamine biosynthesis. After shake-flask fermentation, the engineered strain produced 478.8 mg/L of 1,4-butanediamine within 48 h, representing a 106% increase compared to the control. This study provides a feasible strategy for constructing near-PAMless base editors and lays a foundation for modulating metabolic pathways. Moreover, the engineered strain achieved a marked increase in 1,4-butanediamine production, demonstrating that promoter editing via ABE can effectively regulate metabolic flux and enhance the production of the target product, as well as serving as a valuable reference for the biosynthesis of other high-value-added chemicals.

## 1. Introduction

1,4-Butanediamine (putrescine) is a key monomer for heat-resistant nylon 46, which is widely employed in high-end manufacturing fields like the automotive industry, electronic appliances, and aerospace [1,2,3]. Currently, 1,4-butanediamine is produced industrially mainly via the acrylonitrile-based chemical route, which relies on non-renewable petrochemical feedstocks and involves harsh reaction conditions and significant environmental pollution [4,5]. In contrast, microbial fermentation offers advantages such as renewable raw materials, mild reaction conditions, and environmental benignity, making it a promising alternative for industrial production [6,7].

In *Escherichia coli*, 1,4-butanediamine biosynthesis relies mainly on two endogenous pathways: the ornithine decarboxylase (ODC) pathway and the arginine decarboxylase (ADC) pathway [8]. The ODC pathway and the ADC pathway start with ornithine and arginine, respectively, and lead to the formation of 1,4-butanediamine through dedicated enzymatic conversions. The metabolic network is complex, and the expression levels of various genes require fine-tuning to achieve optimal metabolic flux distribution. Promoters are core elements that regulate gene expression, and altering promoter strength can effectively modulate metabolic flux [9,10,11].

The CRISPR/Cas9-derived base editor can regulate promoter strength by editing promoter sequences, providing an ideal tool for in situ promoter engineering [12,13]. The adenine base editor (ABE) can convert A·T base pairs to G·C, thereby altering promoter strength [14,15]. The most commonly used *Streptococcus pyogenes* Cas9 (SpCas9) strictly relies on the NGG PAM sequence, while NGG sites are scarce in promoter regions, severely limiting the editable range [16,17]. To overcome the NGG limitation, researchers have engineered various Cas9 variants with enhanced PAM compatibility through directed evolution, such as xCas9, SpCas9-NG and SpRY [18,19,20]. Notably, SpRY recognizes PAM sequences encompassing NRN and NYN (where R = A/G, Y = C/T), largely eliminating PAM restriction. Li et al. [21] and Yao et al. [22] employed the near-PAMless SpRY to achieve efficient gene inactivation and functional screening of non-coding sequences at single-base resolution.

**Figure 1:**
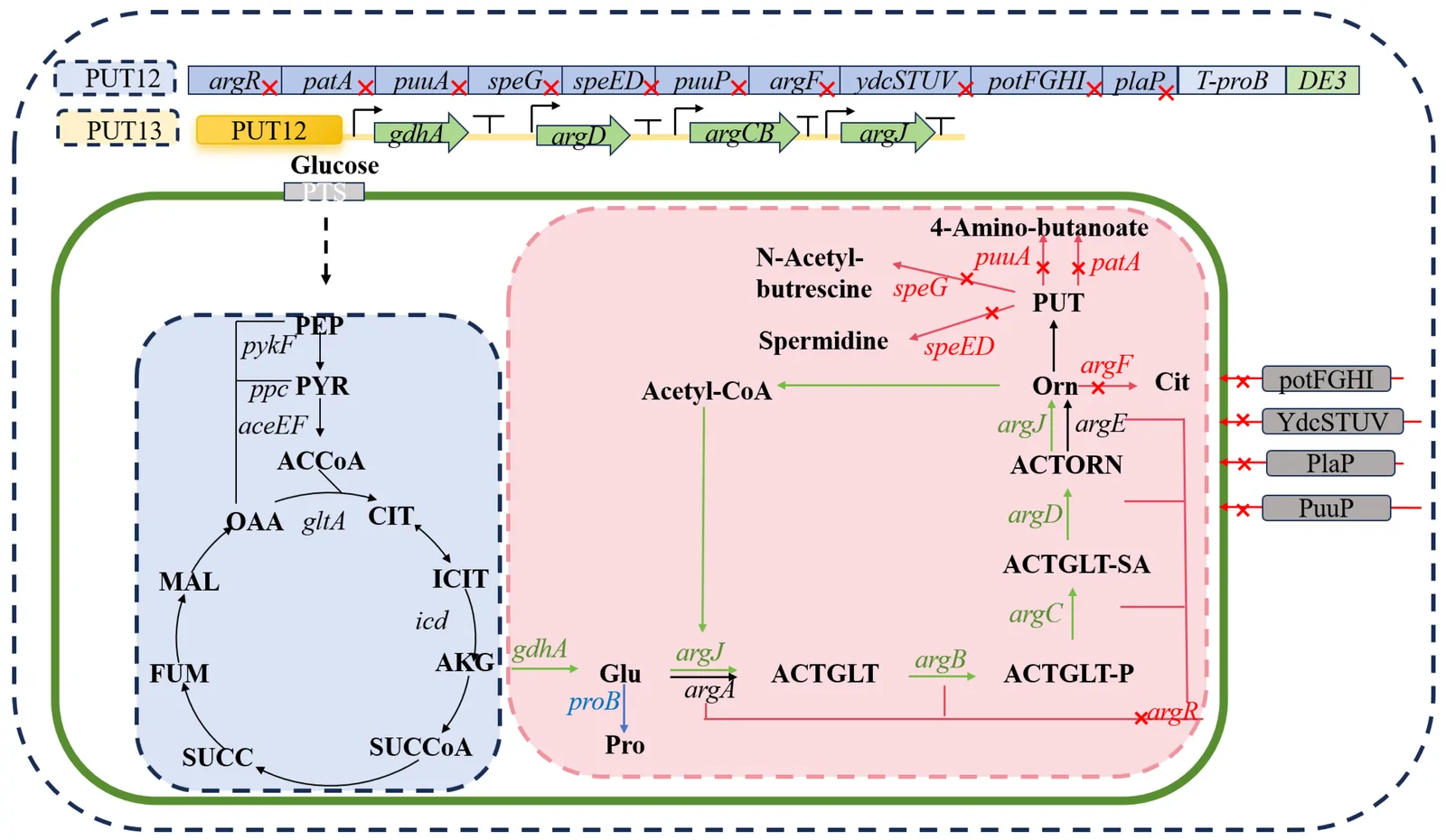
1,4-butanediamine biosynthetic pathway. Genes marked with red crosses (×) indicate knockout targets in the PUT11 strain. Five genes overexpressed in the PUT13 strain (shown in green) are driven by the T7 promoter. Unmodified endogenous genes are shown in black. Blue arrows represent attenuation.

Our laboratory constructed a 1,4-butanediamine-producing chassis strain, PUT11, by knocking out 10 genes and knocking down one gene related to the 1,4-butanediamine metabolic pathway in *E. coli*, including genes involved in degradation, recycling, and intracellular transport of 1,4-butanediamine [23]. Subsequently, the DE3 sequence encoding T7 RNA polymerase was integrated into the PUT11 chromosome to obtain the 1,4-butanediamine-producing strain PUT12. Then, the biosynthetic pathway from glutamate to 1,4-butanediamine was reconstituted by integrating it into PUT12 genome, generating the recombinant strain PUT13 (Figure 1). All genes in the pathway were placed under the control of the strong T7 promoter to enhance metabolic flux. To improve the expression compatibility, we constructed a base editor targeting the T7 promoter.

In this study, we fused the SpRY variant with the high-activity adenine deaminase TadA8e to construct a near-PAMless ABE. This editor was applied to the 1,4-butanediamine-producing strain PUT13 to remodel promoter strength by editing the T7 promoter region, and high-throughput screening was performed using a PuuR transcription factor-based biosensor [24,25], leading to the isolation of high-yield strains. By modulating promoter strength using ABE, this strategy achieves redistribution of metabolic flux and systematic optimization of the metabolic network, providing a new technological route for metabolic engineering.

## 2. Materials and Methods

### 2.1. Strains, Plasmids, and Primers

The strains and plasmids used in this study are listed in Supplementary Table S1. *E. coli* PUT12 and PUT13 were previously constructed in our laboratory. The primers used for construction are listed in Supplementary Table S2.

### 2.2. Construction of the Near-PAMless dCas9RY Variant

To obtain the near-PAMless dCas9RY variant, mutant primers were designed and 13 point mutations (including 11 PAM-widening mutations and 2 catalytic inactivation mutations) were introduced by overlap extension PCR using a Mastercycler^®^ nexus (Eppendorf, Hamburg, Germany). The mutated dCas9RY fragment was cloned into the pRED-Cas9 plasmid between the *Afe*I and *Kpn*I sites using seamless cloning technology, generating the recombinant plasmid pRED-dCas9RY. All mutations were verified by Sanger sequencing, and the construct was used for subsequent experiments.

### 2.3. Construction of the Adenine Base Editor Plasmid

**Figure 2:**
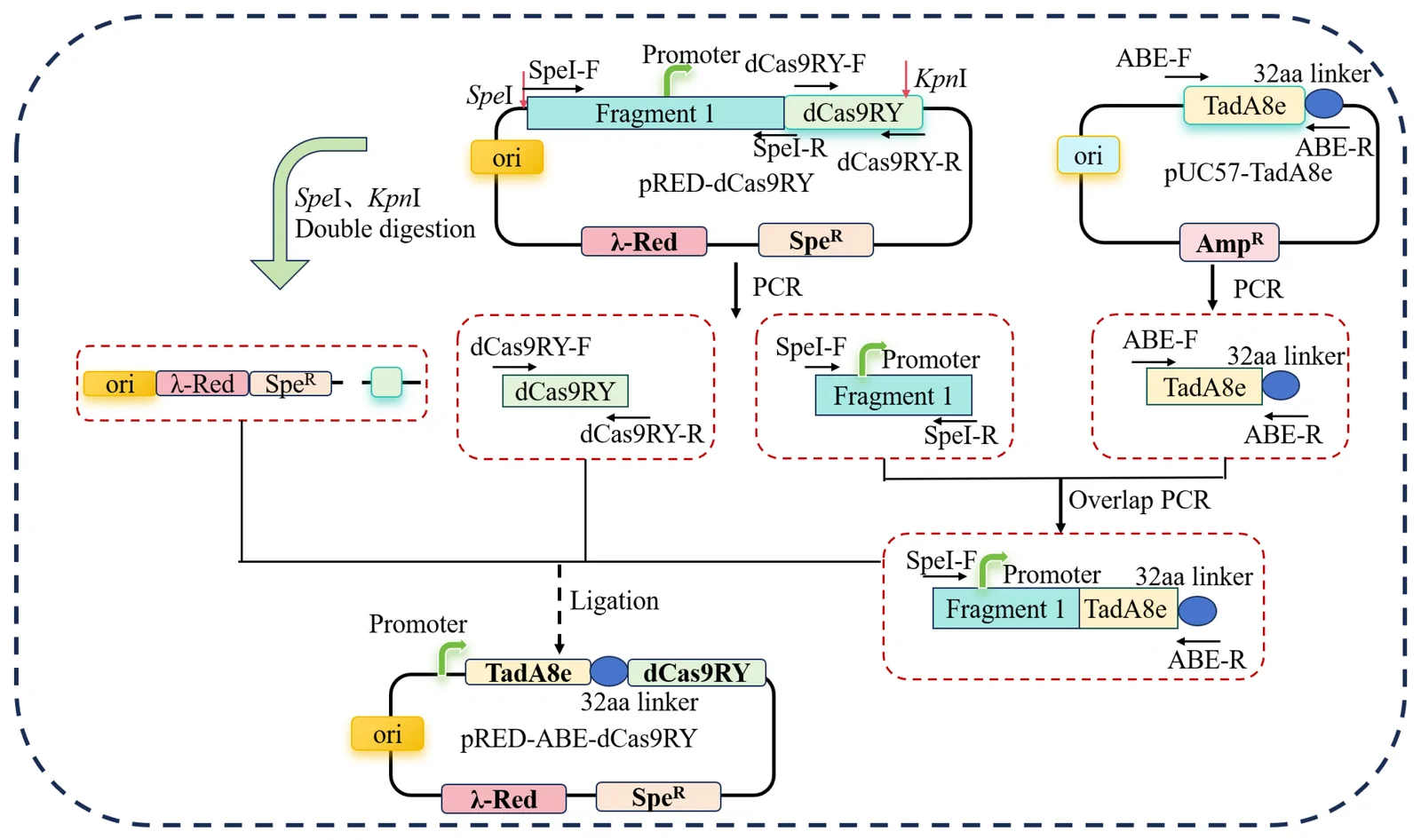
Construction of the pRED-ABE-dCas9RY plasmid. linker: (SGGS)_2_-XTEN-(SGGS)_2_. Fragment 1: The sequence located between the *Spe*I site and the translation initiation site of dCas9RY.

The codon-optimized TadA8e-linker sequence (SGGS)_2_-XTEN-(SGGS)_2_, where XTEN is SGSETPGTSESATPES, was synthesized and cloned into pUC57. The TadA8e-linker fragment and Fragment 1 were fused together by overlap PCR and was then inserted between the *Spe*I and *Kpn*I sites of pRED-dCas9RY along with dCas9RY to generate the recombinant plasmid pRED-ABE-dCas9RY (Figure 2).

### 2.4. Construction of the Plasmids pRED-ABE-dCas9RY-Δλ-Red and pRED-Ptrc*-nCas9RY

The plasmid pRED-ABE-dCas9RY was double-digested with *Apa*I and *Bgl*II to excise the λ-Red recombination system. A 20 bp annealed fragment with cohesive ends of *Apa*I and *Bgl*II was then cloned into the *Apa*I and *Bgl*II sites of the digested vector to construct pRED-ABE-dCas9RY-Δλ-Red, which contains the base editor cassette but lacks the λ-Red system.

A Ptrc*-nCas9RY fragment carrying the constitutive Ptrc* promoter (a Ptrc core promoter variant lacking the lacO operator) and the nCas9RY coding sequence was synthesized and cloned into the *Not*I and *Xho*I sites of the pRED-ABE-dCas9RY-Δλ-Red plasmid to generate the recombinant plasmid pRED-Ptrc*-nCas9RY.

### 2.5. Construction of the Base Editor Targeting Plasmid

**Figure 3:**
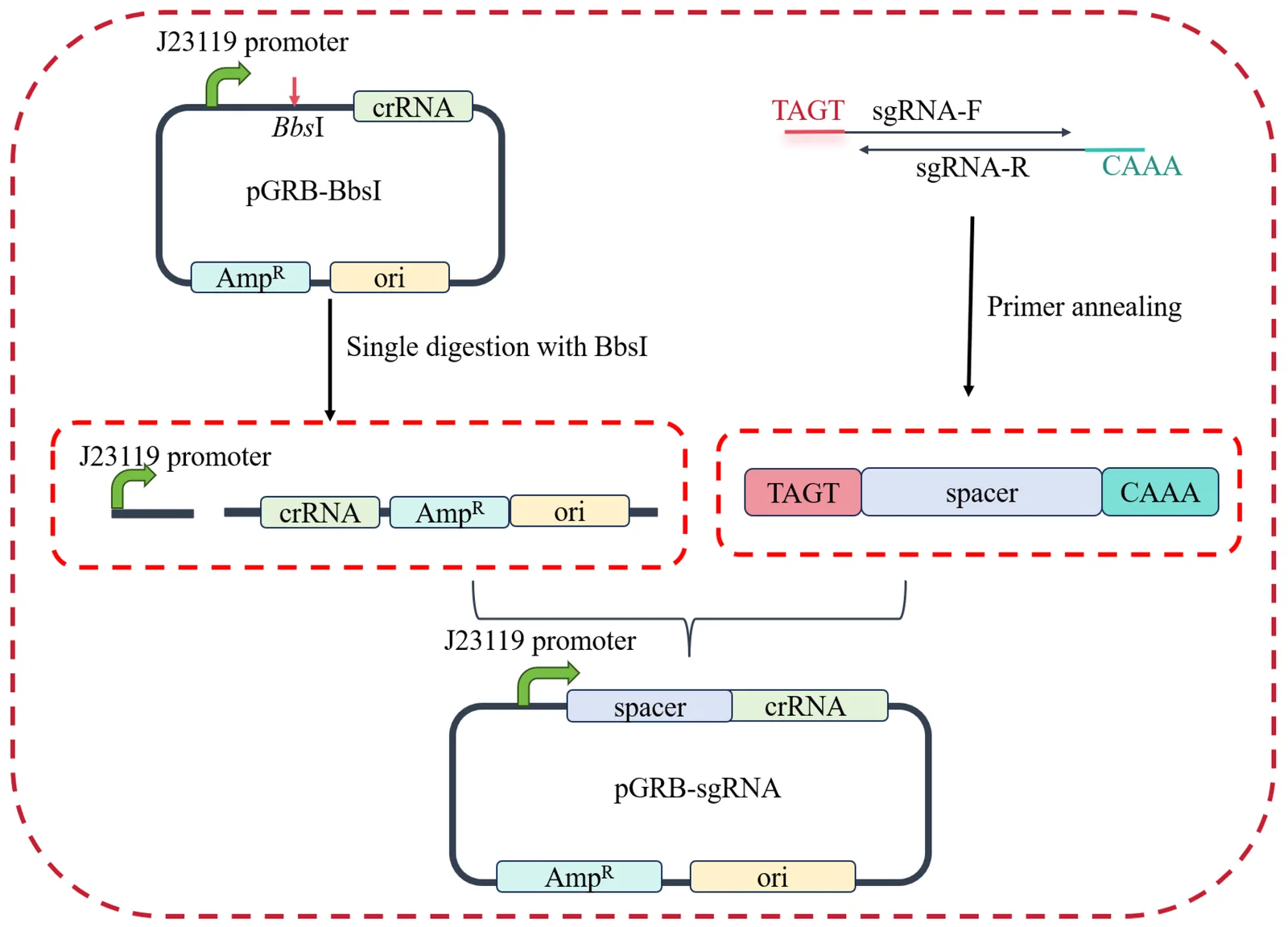
Construction of the pGRB-sgRNA plasmid.

Based on the T7 promoter sequence, a 20 bp sgRNA target sequence was designed using the CRISPOR online tool (http://crispor.tefor.net/). The upstream and downstream primers were synthesized, annealed to form double-stranded DNA, and cloned into the *Bbs*I site of the pGRB plasmid to generate the pGRB-sgRNA plasmid (Figure 3).

### 2.6. Construction of the Base Editor Functional Validation Strain

To validate the function of the base editor, a functional validation strain was constructed by integrating a T7 promoter-driven eGFP reporter cassette into the *pck* locus (encoding phosphoenolpyruvate carboxykinase; a non-essential gene under the conditions used in this study) of the *E. coli* PUT12 genome. First, a sgRNA targeting the *pck* locus was designed and cloned into the pGRB-BbsI vector to obtain the targeting plasmid pGRB-*pck*-sgRNA. Meanwhile, a donor fragment plasmid pUC57-*pck*-up-T7eGFP-dn was constructed, containing approximately 500 bp homology arms upstream and downstream of the *pck* locus and the T7-eGFP expression cassette.

**Figure 4:**
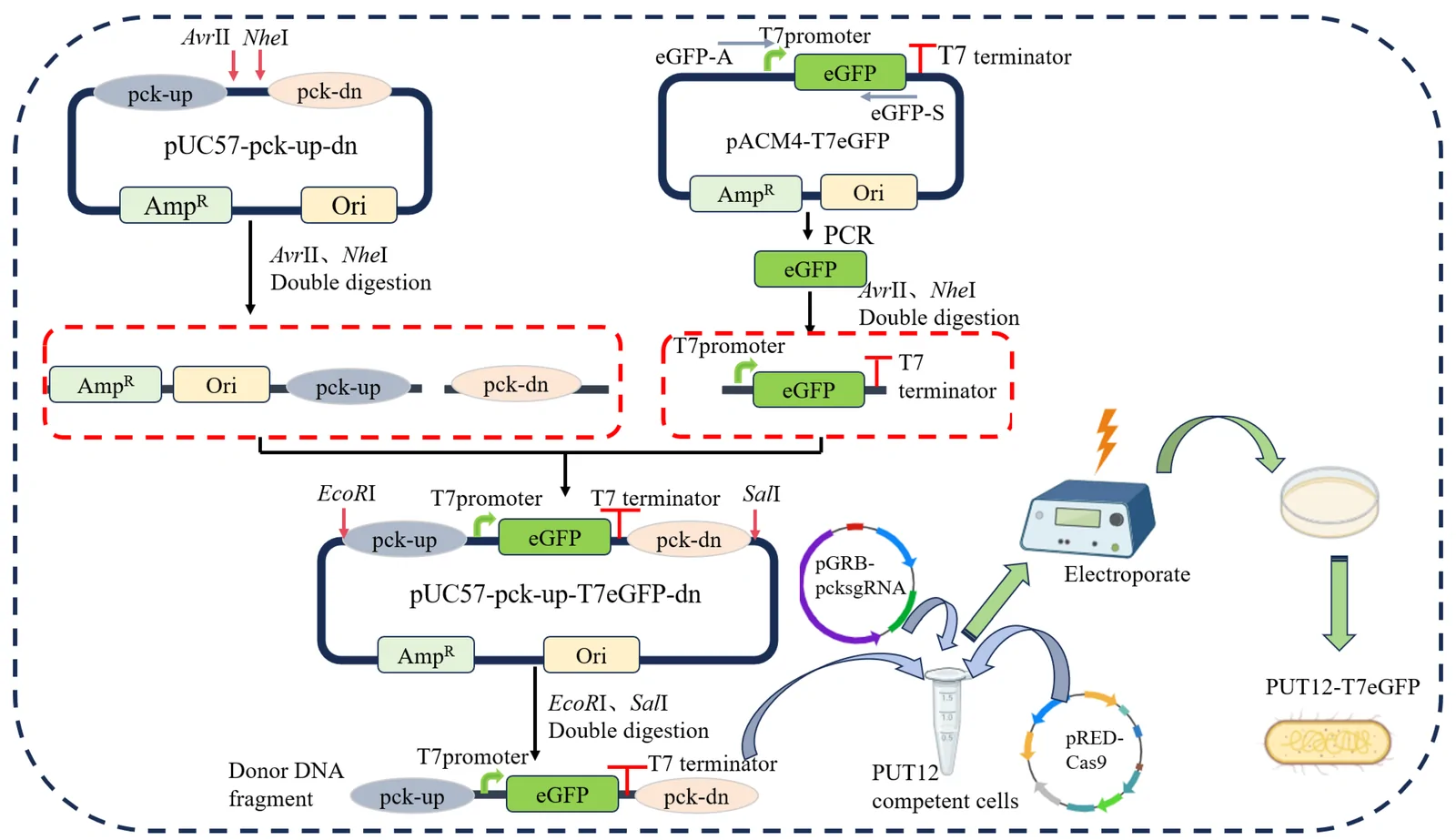
Construction of the PUT12-T7eGFP functional validation strain.

The Cas9-expressing plasmid pRED-Cas9, the targeting plasmid pGRB-*pck*-sgRNA, and the restriction enzyme-linearized donor fragment were co-transformed into PUT12 cells. After transformation, the cells were plated on LB agar plates containing appropriate antibiotics and incubated overnight. Single colonies were picked for colony PCR verification; correctly integrated positive clones were designated as the functional validation strain PUT12-T7eGFP (Figure 4).

### 2.7. Functional Validation of ABE

To validate the function of ABE, the editor plasmid pRED-ABE-dCas9RY and the targeting plasmid pGRB-T7sgRNA were co-transformed into the validation strain PUT12-T7eGFP. The function of ABE was evaluated by measuring changes in eGFP fluorescence intensity.

Transformants were inoculated into 48-well cell culture plates and grown overnight at 32 °C (as the base editor plasmid carries a temperature-sensitive pSC101 origin of replication). Subsequently, the seed culture was transferred (1%, *v*/*v*) into fresh 48-well plates containing 2 mL of LB medium per well, and antibiotics were added to maintain selective pressure. A strain without the base editor plasmid was used as the control. When the cells reached mid-log phase (OD_600_ ≈ 0.6–0.8), IPTG was added to a final concentration of 0.1 mM to induce eGFP expression. After induction at 32 °C for 24 h, the fluorescence intensity was measured using a Tecan Infinite 200PRO microplate reader (Tecan Austria GmbH, Grödig, Austria) with excitation at 488 nm and emission at 523 nm. The editing activity of ABE was assessed by comparing the fluorescence intensity between the experimental group and the control.

### 2.8. Construction of the Recombinant Strain Library and High-Throughput Screening

For high-throughput screening of the recombinant strain, a PuuR-based 1,4-butanediamine biosensor plasmid was used. PuuR is a transcription factor that represses the puu operon in *E. coli*; upon binding of 1,4-butanediamine with PuuR, the repression is relieved, allowing transcription from the *puu* promoter. The biosensor plasmid pRSM3-PuuR-J23119-PgapA-sfGFP(AABC) carries a GFP reporter gene downstream of the PuuR-responsive promoter, such that GFP fluorescence intensity correlates with intracellular 1,4-butanediamine concentration [24]. Therefore, the GFP/OD_600_ ratio was used as a screening indicator to identify high-yield strains. Transformants were inoculated into 48-well plates containing 200 μL of LB medium per well (supplemented with appropriate antibiotics) and cultured at 32 °C, 220 r/min for 24 h in the ZWY-2102C shaker (Shanghai Zhicheng Analytical Instruments Manufacturing Co., Ltd., Shanghai, China). The OD_600_ and GFP fluorescence values were measured, and the GFP/OD_600_ ratio was calculated. A strain transformed with only the biosensor plasmid and the targeting plasmid was used as the control. Candidate strains with markedly higher GFP/OD_600_ ratios compared to the control were selected.

### 2.9. Shake-Flask Fermentation Validation of Candidate Strains

Single colonies of control and recombinant strains were inoculated into 5 mL of liquid LB medium and cultured overnight (12–16 h) at 37 °C with shaking at 220 r/min. The overnight culture was then transferred at 2% (*v*/*v*) into 25 mL of fermentation medium, and the culture incubated until OD_600_ reached approximately 0.8, at which point IPTG was added and cultivation was continued for 48 h. At the end of fermentation (48 h), the 1,4-butanediamine concentration in the broth was determined by HPLC. The fermentation medium consisted of: glucose 30 g/L, (NH_4_)_2_SO_4_ 2 g/L, KH_2_PO_4_ 3 g/L, MgSO_4_·7H_2_O 1 g/L, yeast extract 2 g/L, vitamin B_12_ (VB_12_) 2 mg/L, vitamin B_1_ (VB_1_) 2 mg/L, vitamin B_5_ (VB_5_) 2 mg/L, pyridoxal 5′-phosphate (PLP) 5 mg/L, trace element mixture 5 mL/L, pH 7.0.

### 2.10. Determination of 1,4-Butanediamine Concentration

The concentration of 1,4-butanediamine was determined by o-phthalaldehyde (OPA) pre-column derivatization high-performance liquid chromatography (HPLC) using a commercial OPA reagent according to the manufacturer’s instructions (Agilent Technologies, Inc., Santa Clara, CA, USA). In this derivatization reaction, OPA reacts with primary amines such as 1,4-butanediamine in the presence of a thiol reagent to form highly fluorescent isoindole derivatives, allowing sensitive fluorescence detection by HPLC. The column was an Agilent Eclipse AAA column (4.6 mm × 150 mm). The column temperature was maintained at 40 °C, and the detection wavelength was 338 nm. Mobile phase A was a mixture of Na_2_B_4_O_7_ (2.012 g/L) and Na_2_HPO_4_·2H_2_O (1.78 g/L) adjusted to pH 8.2 with concentrated HCl. Mobile phase B was a mixture of acetonitrile/methanol/water (45:45:10, by volume). The gradient elution program was: 0 min, 2% B; 0.35 min, 2% B; 13.4 min, 57% B; 13.5 min, 100% B; 15.7 min, 100% B; 15.8 min, 2% B; 18.0 min, 2% B. The flow rate was 1 mL/min. Before injection, the fermentation broth supernatant was filtered through a 0.22 μm membrane and automatically injected following the OPA derivatization procedure. The method was validated for 1,4-butanediamine quantification over a linear range of 20–200 mg/L.

### 2.11. Statistical Analysis

For primary screening experiments, each transformant was measured once, and the data were used only for candidate selection rather than for statistical comparison. The shake-flask fermentation validation was performed in three independent biological replicates (n = 3); and data are presented as mean ± standard deviation (SD); figures were generated using OriginPro 2025 (OriginLab Corporation, Northampton, MA, USA). Compared with the control group, a *p* value of 0.01 < *p* < 0.05 indicates a significant difference, and *p* < 0.01 indicates a highly significant difference.

## 3. Results

### 3.1. Construction of a Near-PAMless Cas9 Protein Variant

To overcome the limitation posed by the scarcity of NGG sites in promoter regions for conventional adenine base editors, we first constructed a near-PAMless Cas9 protein variant (dCas9RY) capable of broadly targeting promoter sequences. According to a previous report [20], the Cas9 protein primarily recognizes the canonical NGG PAM sequence through the side chains of R1333 and R1335; systematic mutagenesis of key residues in the PAM-interacting (PI) domain can reshape its PAM recognition preference, thereby yielding a Cas9 variant with broadened PAM compatibility.

To this end, we first introduced the inactivating mutations D10A and H840A in the RuvC and HNH nuclease domains, respectively, to obtain a dCas9 scaffold that retains only target-binding ability. On this basis, we performed site-directed mutagenesis at 11 residues in the PI domain, namely A61R, L1111R, D1135L, S1136W, G1218K, E1219Q, N1317R, A1322R, R1333P, R1335Q and T1337R, to broaden its PAM compatibility. These 13 mutations were sequentially introduced into the pRED-Cas9 plasmid by multiple rounds of overlap extension PCR, yielding the recombinant plasmid pRED-dCas9RY. All mutations were confirmed by Sanger sequencing (Supplementary Figure S1).

### 3.2. Construction of the Base Editor

#### 3.2.1. Construction of the Adenine Base Editor Plasmid

**Figure 5:**
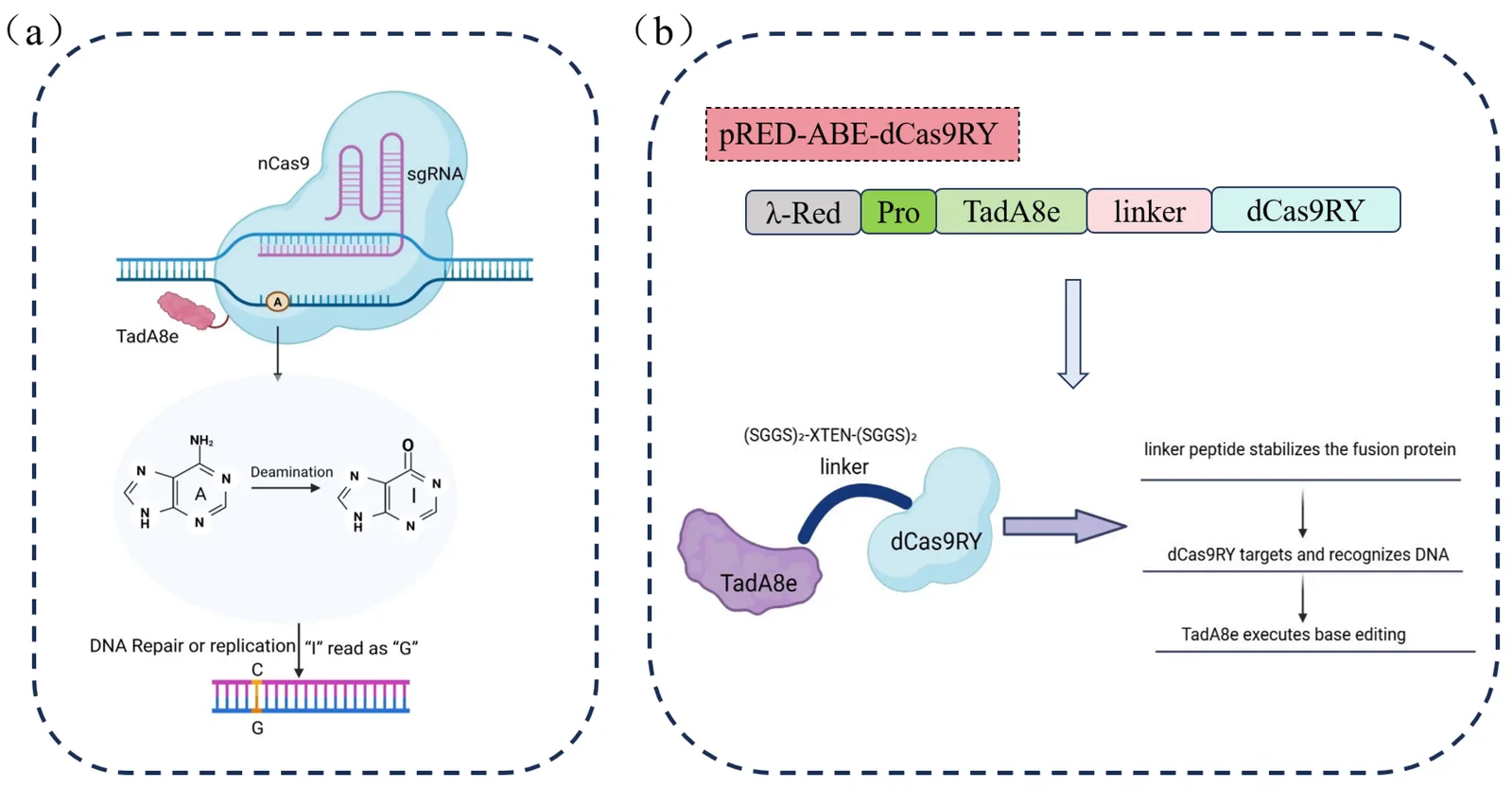
Schematic diagrams and sequencing validation. (**a**) Working principle of the adenine base editor; (**b**) Schematic of the key components of the pRED-ABE-dCas9RY construct. Pro: the original constitutive promoter of the pRED-Cas9 plasmid; λ-Red: derived from bacteriophage λ; TadA8e: highly active adenine deaminase variant evolved from *E. coli* TadA; dCas9RY: catalytically dead SpRY variant carrying the D10A and H840A mutations.

The working principle of the adenine base editor involves fusing an adenine deaminase with a catalytically inactivated Cas9 protein. Under the guidance of sgRNA, it targets a specific genomic site, where the deaminase converts adenine (A) to inosine (I). During DNA replication, inosine is recognized as guanine (G), thereby achieving precise A·T-to-G·C conversion (Figure 5a). Based on this principle, we fused the dCas9RY variant with the adenine deaminase TadA8e using a composite flexible linker composed of SGGS and XTEN in tandem [26], generating the adenine base editor plasmid pRED-ABE-dCas9RY (TadA8e-linker-dCas9RY) (Figure 5b).

#### 3.2.2. Construction of the Base Editor Targeting Plasmid

We designed a specific sgRNA (5′-ATTAATACGACTCACTATAG-3′) targeting the T7 promoter sequence and cloned it into the *Bbs*I site of the pGRB-BbsI vector, generating the sgRNA expression plasmid pGRB-T7sgRNA.

#### 3.2.3. Construction of the Base Editor Functional Validation Strain

**Figure 6:**
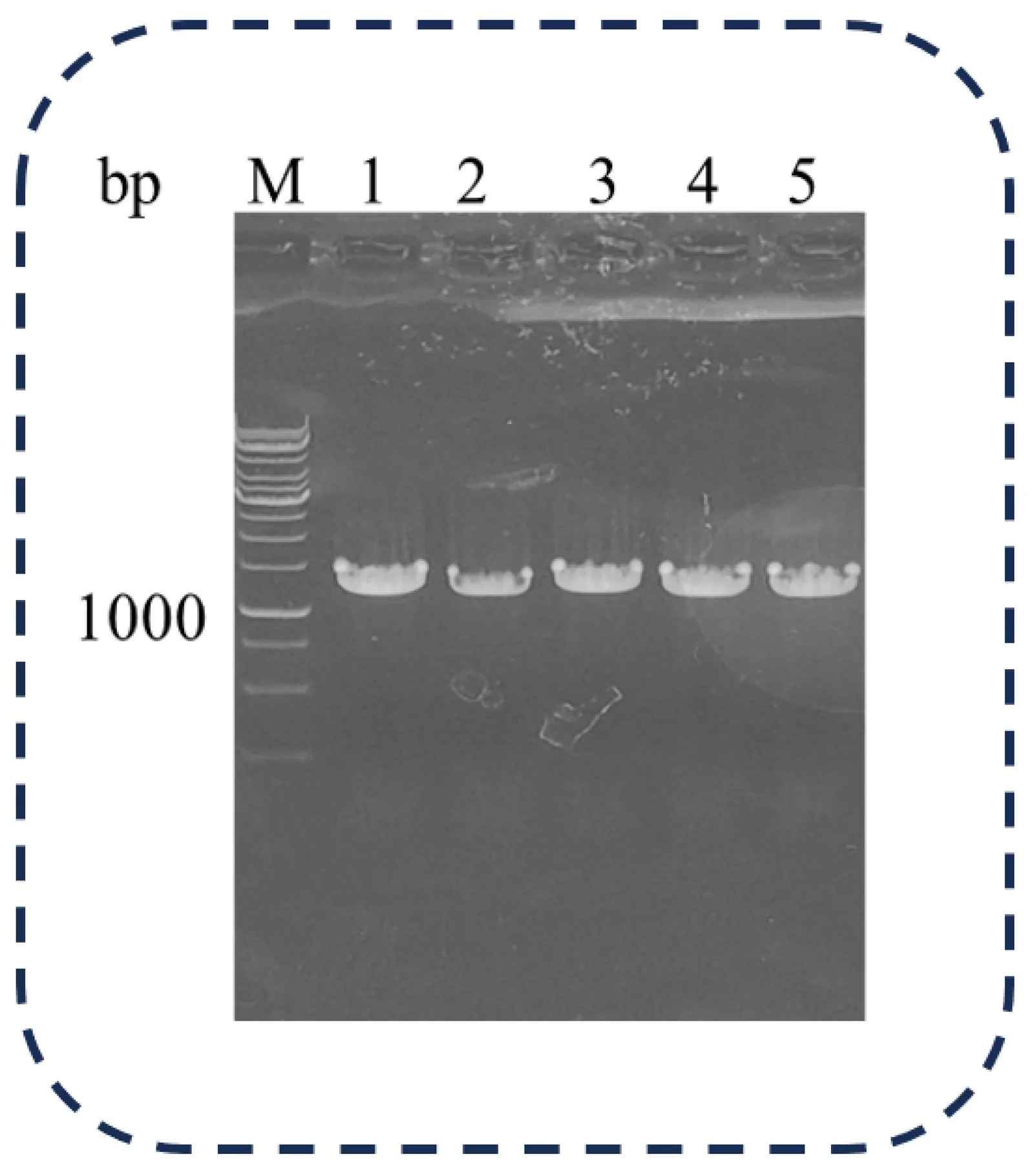
Validation of the recombinant strain PUT12-T7eGFP by colony PCR.

Using the previously constructed 1,4-butanediamine recombinant strain PUT12 as the chassis, we integrated a reporter gene expression cassette into the genomic *pck* locus to generate the functional verification strain PUT12-T7eGFP for the base editor. To this end, the donor plasmid pUC57-*pck*-up-T7eGFP-dn containing the T7-eGFP expression cassette was constructed. After double-restriction enzyme digestion, the fragment containing the upstream and downstream homology arms and the T7-eGFP expression cassette (up-T7eGFP-dn) was recovered and used as a donor fragment for subsequent knock-in experiments. Using CRISPR/Cas9, the donor fragment and the sgRNA expression plasmid pGRB-*pck*-sgRNA (targeting the *pck* locus) were co-electroporated into the PUT12 strain. To verify successful integration, five randomly selected transformants were analyzed by colony PCR, which yielded the expected 1300 bp target band (Figure 6). The knock-in locus of the positive strain was further amplified by PCR and sequenced, and the sequencing results matched the expected sequence. These results indicate that the eGFP expression cassette was successfully integrated into the *pck* locus, and the resulting recombinant strain can be used for subsequent functional validation experiments.

#### 3.2.4. Functional Validation and Result Analysis of the Adenine Base Editor

**Figure 7:**
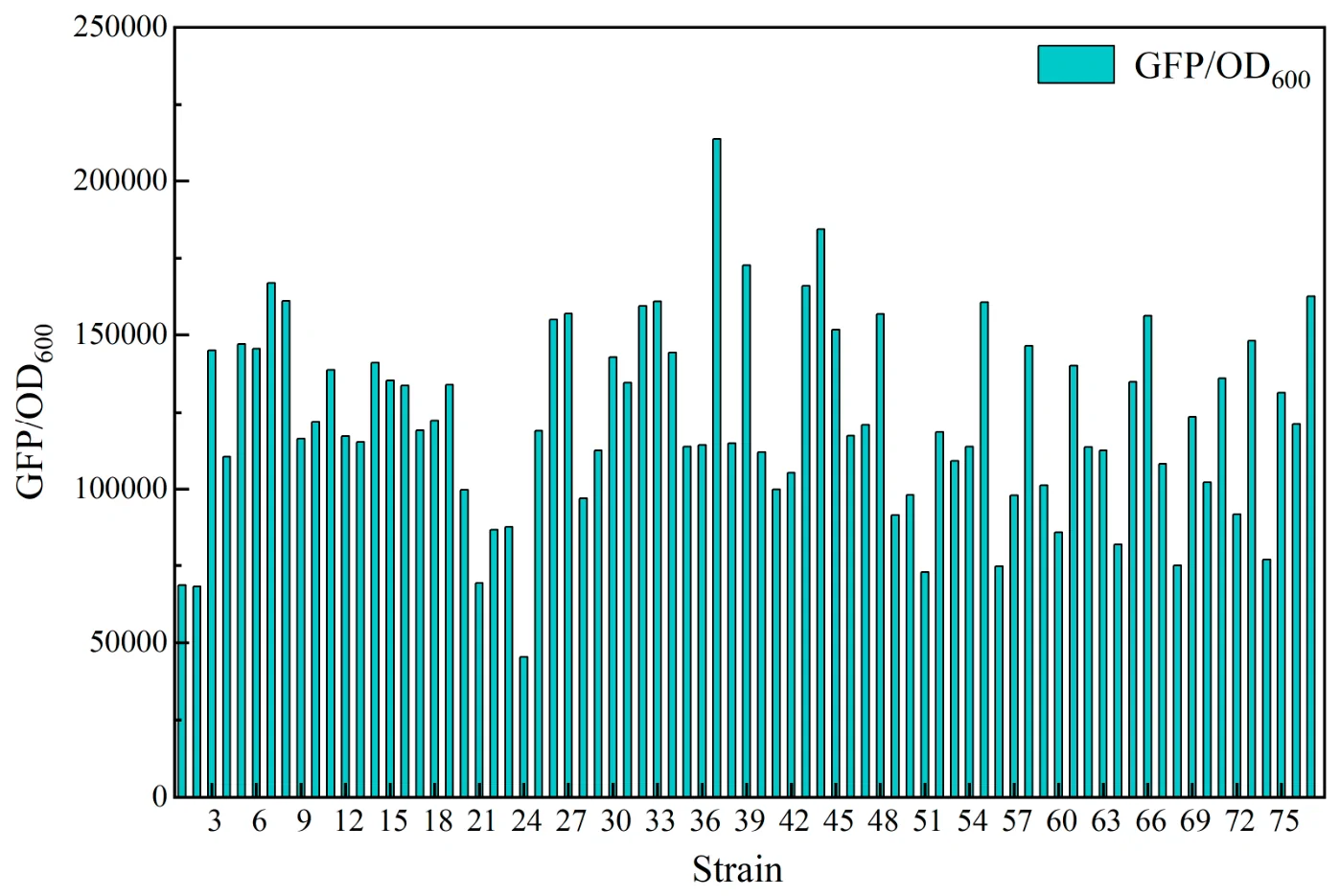
Fluorescence detection results of adenine base editor transformant strains. Shown are the relative eGFP expression levels (GFP/OD_600_ ratio) of 76 transformants (No. 1–76) and the control strain (No. 77) after IPTG induction. Strains were cultured in LB medium with appropriate antibiotics at 32 °C, induced at an OD_600_ of 0.6–0.8, and then measured for fluorescence and OD_600_.

The constructed adenine base editor plasmid pRED-ABE-dCas9RY and the targeting plasmid pGRB-T7sgRNA were co-transformed into the strain PUT12-T7eGFP for functional validation. Seventy-six transformants were randomly selected for fermentation, and the PUT12-T7eGFP strain without the base editor plasmid (No. 77) was used as a control. As shown in Figure 7, strains No. 24 and No. 37 exhibited markedly different GFP/OD_600_ ratios compared with the control strain. However, PCR amplification and sequencing of the target sites of these strains revealed that no base change occurred in the T7 promoter region, indicating that the adenine base editor did not display detectable targeted editing activity under the tested conditions. The possible reasons were the CRISPRi effects [27,28] by the binding of sgRNA-guided TadA8e-linker-dCas9RY to T7 promoter and interference from the λ-Red recombination system.

### 3.3. Optimization of the Base Editor

To obtain an active base editor, we performed multiple rounds of optimization, including removal of the λ-Red system, replacement of dCas9RY with nCas9RY, codon optimization, substitution of the promoter with Ptrc*, and redesign of the sgRNA.

#### 3.3.1. Multiple Optimization of the Base Editor Plasmid

**Figure 8:**
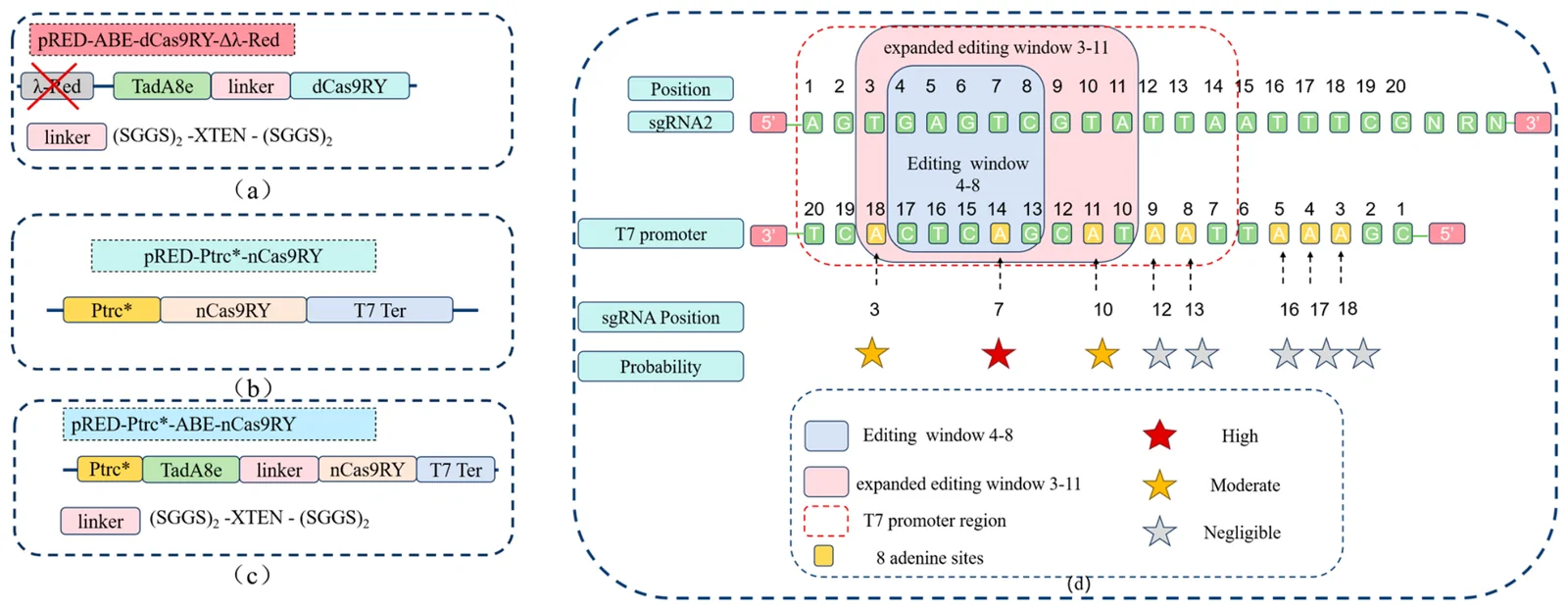
Schematic diagrams of the key components of base editor constructs and the sgRNA target sequence. (**a**) pRED-ABE-dCas9RY-Δλ-Red construct, in which the λ-Red recombination system was deleted; (**b**) pRED-Ptrc*-nCas9RY construct, in which a constitutive Ptrc* promoter drives transcription of the nCas9RY protein (SpRY variant with D10A mutation); (**c**) pRED-Ptrc*-ABE-nCas9RY construct, in which the Ptrc* constitutive promoter drives transcription of the adenine deaminase variant TadA8e and nCas9RY; (**d**) schematic of the sgRNA2 target site and editing window analysis. The core editing window of TadA8e spans positions 4–8 relative to the PAM (blue box), with an extended range of 3–11 (pink box). The color gradient indicates editing probability, with darker shading representing higher probability, and the efficiency is highest at the center and decreases toward the edges.

To eliminate the potential impact of the λ-Red recombination system on base editing, this system was deleted from the adenine base editor plasmid pRED-ABE-dCas9RY, yielding the plasmid pRED-ABE-dCas9RY-Δλ-Red (Figure 8a).

To enhance the expression level of the Cas9 variant, we optimized the coding sequence of dCas9RY based on the codon usage bias of *E. coli*. In addition, to further increase base editing efficiency, we converted the protein into a nickase (nCas9RY) by introducing the D10A mutation while preserving the 11 mutations that expand PAM recognition, because nCas9 specifically nicks the non-edited strand, prompting the cell to engage long-patch or mismatch repair pathways. As a result, the cell preferentially uses the edited strand as a repair template, thereby fixing the editing event and enhancing overall efficiency [12]. To enhance the expression level of nCas9RY, we placed it under the control of the strong constitutive promoter Ptrc*. The designed Ptrc*-nCas9RY sequence was synthesized and cloned into the pRED-ABE-dCas9RY-Δλ-Red plasmid at the *Not*I and *Xho*I sites, generating the plasmid pRED-Ptrc*-nCas9RY (Figure 8b). Subsequently, the highly active adenine deaminase variant TadA8e was fused to the N-terminus of nCas9RY via a flexible linker, yielding the final adenine base editor plasmid pRED-Ptrc*-ABE-nCas9RY (Figure 8c), in which the entire TadA8e-linker-nCas9RY fusion cassette is transcriptionally driven by the constitutive Ptrc* promoter.

#### 3.3.2. Optimization of the sgRNA Target Sequence of the Base Editor Targeting Plasmid

The promoter sequence was re-analyzed using the CRISPOR online tool (http://crispor.tefor.net/) to identify an sgRNA target sequence with a higher predicted targeting efficiency and a lower predicted off-target score, designated sgRNA2 (5′-AGTGAGTCGTATTAATTTCG-3′) (Figure 8d). This sgRNA target sequence was then cloned into the *Bbs*I site of plasmid pGRB-BbsI to construct plasmid pGRB-T7sgRNA2, which targets the T7 promoter. The core editing window of TadA8e spans positions 4–8 relative to the PAM, and can be extended to positions 3–11 [14,29], where the editing activity peaks at the central region (positions 4–8) and progressively declines toward the periphery (position 3 and positions 9–11).

#### 3.3.3. Functional Validation of the Adenine Base Editor

**Figure 9:**
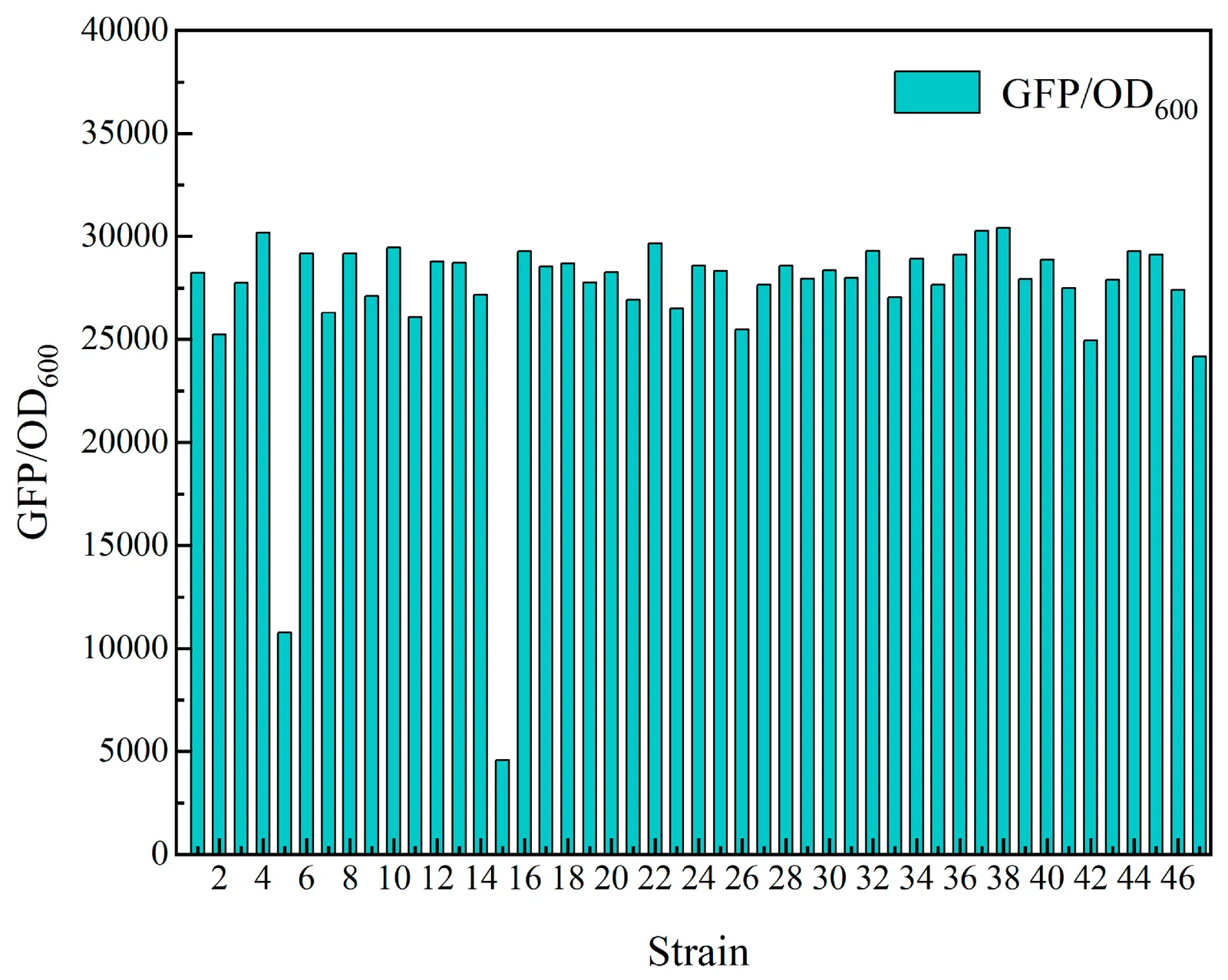
Fluorescence detection results of adenine base editor transformant strains. Shown are the relative eGFP expression levels (GFP/OD_600_ ratio) of 46 transformants (No. 1–46) and the control strain (No. 47) after IPTG induction. Strains were cultured in LB medium with appropriate antibiotics at 32 °C, induced at an OD_600_ of 0.6–0.8, and then measured for fluorescence and OD_600_.

The adenine base editor plasmid pRED-Ptrc*-ABE-nCas9RY and the targeting plasmid pGRB-T7sgRNA2 were co-transformed into the functional validation strain PUT12-T7eGFP. Forty-six transformants were randomly picked and cultured in 48-well plates, and the PUT12-T7eGFP strain without the base editor plasmid (designated as No. 47) was used as a control. As shown in Figure 9, the GFP/OD_600_ ratios of strains No. 5 and No. 15 differed markedly from that of the control strain (No. 47). Therefore, these two transformants were selected for further verification.

**Figure 10:**
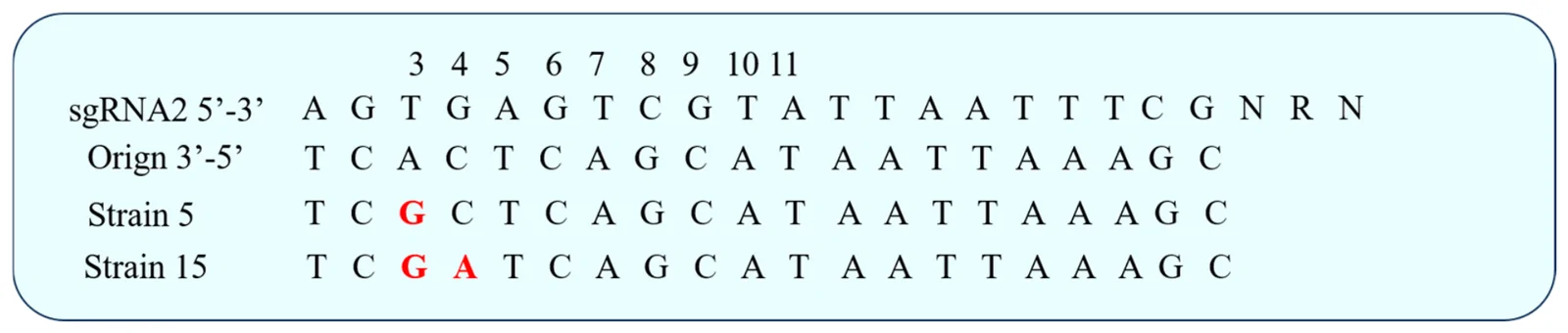
Base editing sites in different strains. Strain No. 5, showing a single A → G edit at position A3 (within the expanded editing window of TadA8e); Strain No. 15, showing an A → G edit at position A3 and an unintended C → A substitution at position C4.

The target regions of strains No. 5 and No. 15 were amplified by PCR and subjected to sequencing. The editing window of TadA8e is defined as positions 4–8 relative to the PAM, although TadA8e is also known to exhibit a broader editing window extending to positions 3–11 [14,29]. As shown in Figure 10, strain No. 5 carried a single A → G edit at position A3 (within the expanded editing window). Strain No. 15 contained an intended A → G edit at the same position, as well as an unintended C → A substitution at position C4 (Figure 10). It has been reported that TadA8e frequently introduces bystander edits when targeting specific adenines, including unintended editing of cytosine (C) [30]. Although such bystander editing reduces the precision of the editor, it simultaneously increases the diversity of the promoter mutation library, which may be beneficial for screening promoter variants with different strengths.

### 3.4. Application of the Base Editor for Modulating the 1,4-Butanediamine Biosynthetic Pathway

To regulate the 1,4-butanediamine biosynthetic pathway, we transformed the adenine base editor into the recombinant strain PUT13. This strain harbors a synthetically assembled pathway from glutamate to 1,4-butanediamine, in which all key enzyme genes are driven by the T7 promoter. By editing the T7 promoter to modulate its strength, we achieved regulation of 1,4-butanediamine biosynthesis, aiming to obtain high-yield strains with improved fitness.

#### 3.4.1. Construction of Recombinant Strains and Preliminary Screening

**Figure 11:**
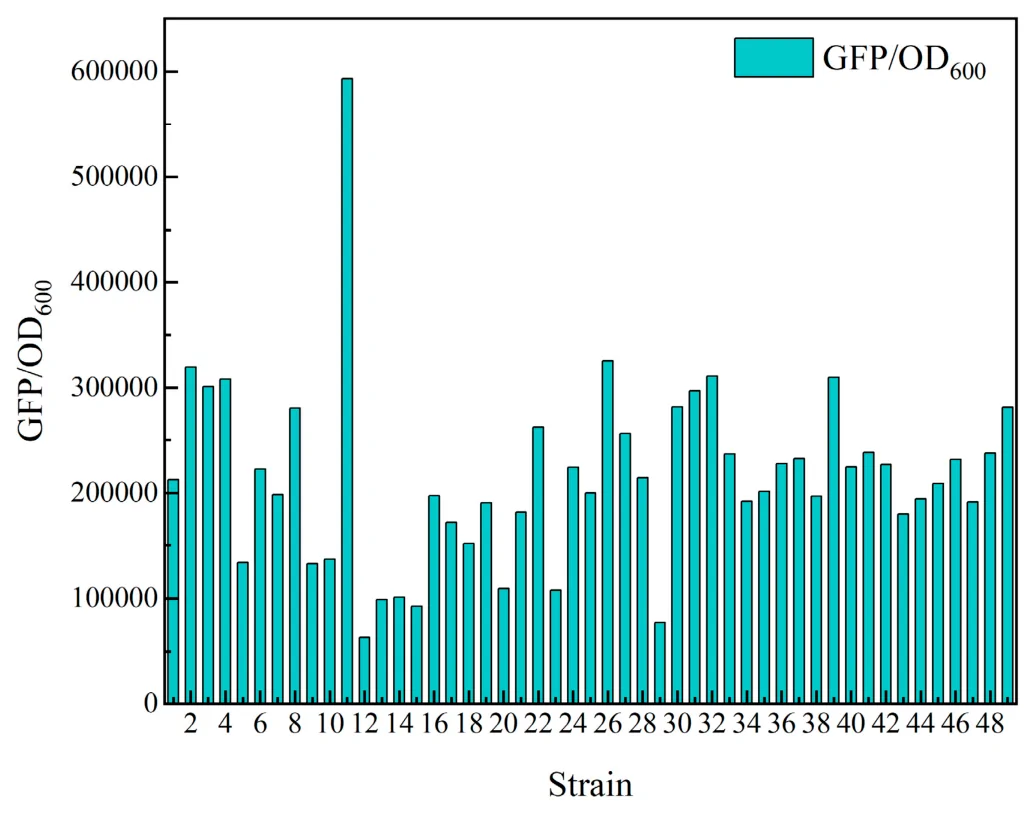
Fluorescence detection results of mutant library recombinant strains in primary screening. Shown are the relative eGFP expression levels (GFP/OD_600_ ratio) of 48 transformants (No. 1–48) and the control strain (No. 49) after IPTG induction. Strains were cultured in LB medium with appropriate antibiotics at 37 °C, induced at an OD_600_ of 0.6–0.8, and then measured for fluorescence and OD_600_.

The biosensor plasmid, the adenine base editor plasmid pRED-Ptrc*-ABE-nCas9RY, and the targeting plasmid pGRB-T7sgRNA2 were co-transformed into strain PUT13. Through ABE-mediated editing of the T7 promoter, a library of recombinant strains with modulated 1,4-butanediamine biosynthetic pathways was constructed, and high-throughput screening was performed using the biosensor. This biosensor, previously constructed in our laboratory based on the transcription factor PuuR, exhibits a fluorescence signal intensity that is positively correlated with 1,4-butanediamine production [24]. Transformants were randomly picked and inoculated into 48-well plates for preliminary screening. After 24 h of fermentation, the GFP/OD_600_ ratio was measured, and candidate strains with markedly higher fluorescence values were selected. As shown in Figure 11, a total of 48 transformants were picked; strain No. 49, which received only the biosensor plasmid and the targeting plasmid, served as the control (PUT13-K). Compared with the control, strain No. 11 exhibited a markedly higher GFP/OD_600_ ratio, which was 2.22-fold that of the control; strains No. 2 and No. 26 were slightly higher; and strains No. 12 and No. 29 were markedly lower. These results suggest that the base editor may be able to edit the T7 promoter, resulting in a redistribution of metabolic flux, yielding recombinant strains with different levels of 1,4-butanediamine production.

#### 3.4.2. Secondary Screening and Yield Analysis of Recombinant Strains

**Figure 12:**
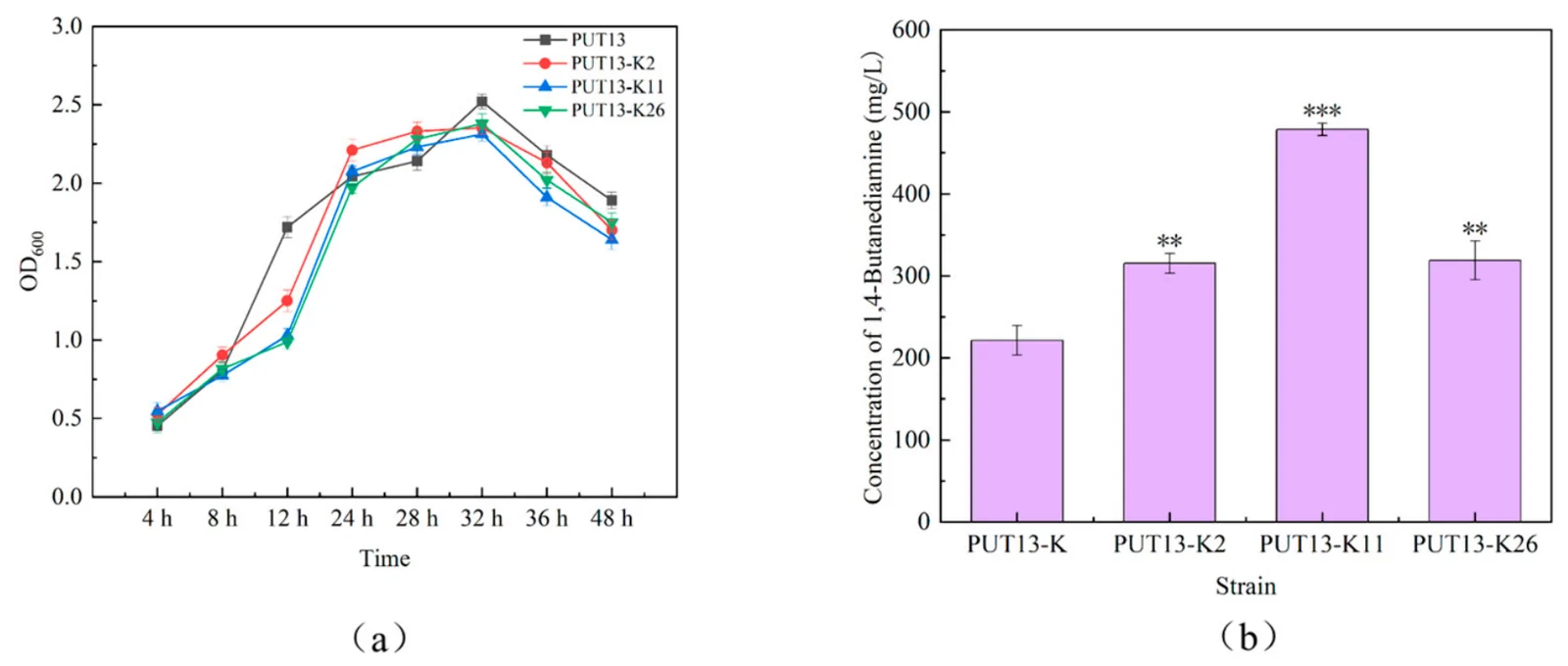
(**a**) Growth curves of recombinant strains. (**b**) Quantification of 1,4-butanediamine produced by recombinant strains. Samples were taken at the end of fermentation (48 h) for metabolite quantification. Data are expressed as means, and error bars indicated standard deviation (n = 3 independent experiments. The ** symbol represented *p* < 0.01 and *** represented *p* < 0.001 compared with the control).

The strains No. 2, No. 11 and No. 26 obtained from the preliminary screening were cured of plasmids and designated PUT13-K2, PUT13-K11 and PUT13-K26, respectively. They were then cultured in shake flasks, and the production of 1,4-butanediamine was measured. The results are shown in Figure 12. As can be seen from Figure 12a, the strains entered the logarithmic growth phase at 4 h and reached the death phase at 32 h, with essentially the same growth pattern. From Figure 12b, the 1,4-butanediamine titers of PUT13-K11, PUT13-K2 and PUT13-K26 were 478.8, 325.8 and 344.6 mg/L, respectively, representing increases of 106%, 40.3% and 48.4% compared with the control strain PUT13-K. These results demonstrate that editing the promoter with the base editor can effectively regulate the 1,4-butanediamine biosynthetic pathway and achieve redistribution of metabolic flux, thereby validating the feasibility and effectiveness of the base editor for modulation of metabolic pathways and metabolic engineering.

**Figure 13:**
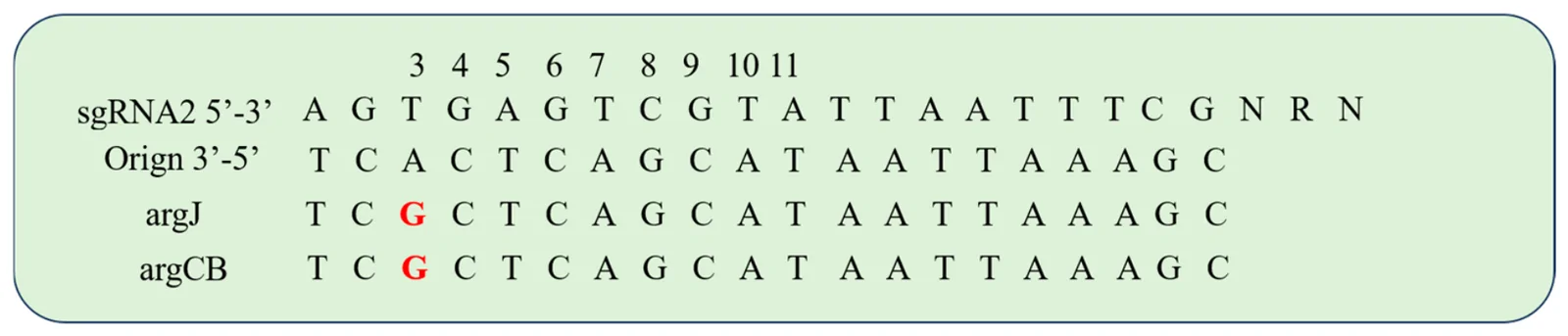
Base editing sites in the promoter regions of *argCB* and *argJ.*

To verify whether the PUT13-K11 promoter was edited, we PCR-amplified the T7 promoter target region and performed sequencing analysis. The sequencing results, as shown in Figure 13, revealed A→G conversions in the T7 promoter regions of both *arg*J and *arg*CB, indicating that the base editor can modulate metabolic flux through promoter editing.

## 4. Discussion

Tuning promoter strength is an effective strategy for optimizing metabolic flux distribution in metabolic engineering [9,10,11]. To achieve this, we constructed a base editor targeting the T7 promoter, which allowed us to modulate promoter strength in the recombinant strain PUT13. As a result, we successfully redistributed the biosynthetic flux of putrescine and increased its production.

Base editors have been employed for in situ modulation of multigene expression. For example, the BETTER (Base Editor-Targeted and Template-free Expression Regulation) strategy [31] employs CRISPR-guided base editors to generate a large number of genetic combinations in situ without relying on donor DNA, enabling the diversification of ribosome binding sites, 5′ untranslated regions, or promoters. In addition, a PAM-less base editing toolbox based on the SpRY variant has been established in *Bacillus subtilis* [31], enabling in situ base editing of nearly all adenines and cytosines across the genome and demonstrating great potential for metabolic engineering and codon expansion. In this study, we achieved modulation of metabolic flux in the 1,4-butanediamine biosynthetic pathway through in situ editing of the T7 promoter, providing a feasible strategy for efficient synthesis of the target product.

During the initial functional validation, certain strains (e.g., strains 24 and 37) exhibited substantial changes in GFP/OD_600_ fluorescence, yet sequencing revealed no mutations in the promoter region. These changes in reporter gene fluorescence may primarily arise from the CRISPRi effect [27,28], wherein the nCas9RY-sgRNA complex binds to the T7 promoter region and physically hinders RNA polymerase transcription elongation. However, other contributing factors, such as interference from the λ-Red recombination system, the intrinsic properties of the deaminase, its expression level, and sgRNA efficiency, cannot be excluded. To address these possibilities, we systematically optimized the editing system and ultimately constructed a promoter-targeted ABE capable of introducing both A → G and C → A conversions within the promoter region.

It should be noted that, during functional validation, we selected only those strains exhibiting significant GFP/OD_600_ changes for promoter sequencing, rather than performing random sequencing. While this approach reduced the workload, it precludes the calculation of ABE editing efficiency. This limitation will be addressed in our future studies.

Traditional promoter library construction methods [32] allow for the screening of promoters with different strengths, which has certain significance for tailoring gene expression and distributing metabolic flux in metabolic pathways. However, they often fail to achieve ideal gene expression compatibility and optimal flux allocation. In this context, in situ promoter editing combined with high-throughput screening can help obtain recombinant strains with fully balanced metabolic flux and increase the production of target products. To this end, we constructed a base editor and obtained recombinant strains with increased 1,4-diaminobutane production through high-throughput screening. Nevertheless, the production levels obtained in this study were still lower than the highest values reported in the literature [6,33,34]. This is mainly because our experimental objective was to verify the feasibility of using a base editor to regulate promoter strength and thereby redistribute metabolic flux, rather than to pursue maximum yields. The recombinant strain used in this study was subjected only to simple modifications for proof-of-concept purposes and still lacks comprehensive pathway engineering as well as optimization of energy and cofactor balances.

Importantly, sequencing of the promoter region in the recombinant strain PUT13-K11 confirmed that the constructed ABE successfully achieved promoter editing, redistribution of metabolic flux, and increased production of 1,4-diaminobutane. These results not only validate the feasibility of our strategy but also provide a theoretical basis and technical reference for editing other promoters and reprogramming metabolic flux in future studies.

Off-target effects of base editors are primarily associated with the spacer sequence of the sgRNA, the activity of the deaminase, and the performance of nCas9RY. Since the spacer sequence selected in this study did not exhibit any highly similar sequences in the genome of the host strain, we did not assess the off-target effects of the ABE in this study. In subsequent strain construction efforts, we will perform whole-genome sequencing to detect off-target effects and alterations in the complete metabolic pathway.

In summary, this study demonstrates that the near-PAMless ABE, combined with biosensor-based high-throughput screening, can effectively edit promoters and modulate metabolic flux, thereby providing a new strategy for improving the production of target compounds. Although the current editing efficiency and product titers still have considerable room for improvement, and limitations remain in mutation identification, off-target analysis, and flux validation, this work provides a proof-of-concept for the application of near-PAMless base editors in prokaryotic promoter engineering and offers a reference for strain improvement of other bio-based chemicals.

## 5. Conclusions

In this study, we successfully constructed an adenine base editor (ABE) that efficiently recognizes and edits the T7 promoter region, achieving A → G conversions and bystander C → A substitutions. Through in situ editing of the T7 promoter, multiple promoter strength variants were generated, altering the expression levels of pathway genes and enabling redistribution of metabolic flux in the 1,4-butanediamine biosynthetic pathway. This study demonstrates that base editors can serve as effective tools for in situ promoter engineering in metabolic engineering, providing technical support and a strategic reference for the optimization of metabolic networks and product synthesis in industrial strain development.

## Data Availability

The raw data supporting the conclusions of this article will be made available by the authors on request.

## Supplementary Materials

The following supporting information can be downloaded at: https://www.mdpi.com/article/10.3390/microorganisms14081708/s1. Table S1: Strains and plasmids used in this study. Table S2: Primers used in this study. Supplementary Figure S1: Sequencing chromatograms of the mutation sites..
