# Supplementary material for: Constructing an Adenine Base Editor in Escherichia coli for Fine-Tuning the 1,4-Butanediamine Biosynthetic Pathway
Source: Microorganisms. 2026 Aug 4;14(8):1708. doi: 10.3390/microorganisms14081708 (PMC13515164; doi:10.3390/microorganisms14081708)
Supplement: Supplementary file 1 [file microorganisms-14-01708-s001.zip › microorganisms-4398552-supplementary.pdf]

Supplementary Table S1: Strains and plasmids used in this study.

Strain/Plasmid	Description	Source
JM109	*E. coli* strain, host for plasmid construction	Stored in our laboratory
K12	*E. coli* strain	Stored in our laboratory
PUT12	Modified *E. coli* K12 strain, host for base editor expression	Stored in our laboratory
pRED-Cas9	Spe^R, harbouring Cas9 protein	Stored in our laboratory
pUC57-pck-up-dn	Amp^R, harboring the upstream and downstream homology arm fragments of the *pck* gene	Stored in our laboratory
pRSM3-PuuR-J23119-PgapA-sfGFP(AABC)	Biosensor plasmid	Stored in our laboratory
PUT12-T7eGFP	Base editor expression host with integrated eGFP expression cassette	This study
pACM4-T7eGFP	Cam^R, T7 promoter-driven eGFP	This study
pRED-Cas9-1	Spe^R, carrying Cas9 protein with D10A mutation	This study
pRED-Cas9-2	Spe^R, carrying Cas9 protein with D10A and A61R mutations	This study
pRED-Cas9-3	Spe^R, carrying Cas9 protein with D10A, H840A and A61R mutations	This study
pRED-Cas9-4	Spe^R, Carrying Cas9 protein with D10A, H840A, A61R and L1111R mutations	This study
pRED-Cas9-5	Spe^R, Carrying Cas9 protein with D10A, H840A, A61R, L1111R, D1135L and S1136W mutations	This study
pRED-Cas9-6	Spe^R, Carrying Cas9 protein with D10A, H840A, A61R, L1111R, D1135L, S1136W, G1218K and E1219Q mutations	This study
pRED-Cas9-7	Spe^R, Carrying Cas9 protein with D10A, H840A, A61R, L1111R, D1135L, S1136W, G1218K, E1219Q, N1317R and A1322R mutations	This study
pRED-dCas9RY	Spe^R, Carrying dCas9RY variant with D10A, H840A, A61R, L1111R, D1135L, S1136W, G1218K, E1219Q, N1317R, A1322R, R1333P, R1335Q and T1337R mutations	This study
pRED-ABE-dCas9RY	Spe^R, Carrying dCas9RY variant and adenine deaminase variant (TadA8e)	This study
pRED-ABE-dCas9RY-Δλ-Red	Spe^R, Carrying dCas9RY variant and adenine deaminase, Δλ-Red	This study
pRED-Ptrc-ABE-nCas9RY	Spe^R, The Ptrc constitutive promoter drives transcription of the nCas9RY variant and TadA8e	This study
pRED-Ptrc-nCas9RY	Spe^R, Ptrc constitutive promoter drives transcription of nCas9RY variant	This study

pGRB-T7sgRNA	Amp^R, expression vector for sgRNA targeting the T7 promoter	This study
pGRB-T7sgRNA2	Amp^R, expression vector for sgRNA targeting the T7 promoter	This study
pUC57-pck-T7eGFP-up-dn	Amp^R, carrying the upstream and downstream homology arm fragments of the *pck* gene and the T7 promoter-driven eGFP expression cassette	This study
pUC57-TadA8e	Carrying the gene sequence of TadA8e	Synthesized by Jiutian Gene Company

Supplementary Table S2: Primers used in this study.

Primer	Sequence（5′–3′）
D10A-F	TACTCAATAGGCTTAGCAATCGGCACAAATAGC
D10A-R	GCTATTTGTGCCGATTGCTAAGCCTATTGAGTA
A61R-F	GGAGAGACAGCGGAACGTACTCGTCTCAAACGG
A61R-R	CCGTTTGAGACGAGTACGTTCCGCTGTCTCTCC
H840A-F	GATTATGATGTCGATGCCATTGTTCCACAAAGT
H840A-R	ACTTTGTGGAACAATGGCATCGACATCATAATC
L1111R-F	TCTCCAAGGAGTCAATTCGCCCGAAAAGAAATTCGGACAA
L1111R-R	TTGTCCGAATTTCTTTTCGGGCGAATTGACTCCTTGGAGA
D1135-1136-F	GATCCAAAAAAATATGGTGGTTTTCTGTGGCCAACGGTAGCTTATTCAGTCC
D1135-1136-R	GGCTGAATAAGCTACCGTTGGCCACAGAAAACCACCATATTTTTTTGGATC
G1218-1219-F	GGATGCTGGCTAGTGCCAAACAGTTACAAAAAGGAAATGAGCTGGC
G1218-1219-R	GCCAGCTCATTTCCTTTTTGTAACTGTTTGGCACTAGCCAGCATCC
N1317-1322-F	CGTTGACGCGTCTTGGAGCTCCCCGTGCTTTTAAATATTTTGATACAACAATT
N1317-1322-R	TATTTAAAAGCACGGGGAGCTCCAAGACGCGTCAACGTAAATAAAT
1333-1335-1337-F	TTTGATACAACAATTGATCCTAAACAATATCGGTCTACAAAAGAAGTTT
1333-1335-1337-R	GTAGACCGATATTGTTTAGGATCAATTGTTGTATCAAAATA
SpeI-F	GGGCTAGTCAATGATAATTACTAGTCCTTTTC
SpeI-R	TCTGACATTTTTGCCTCCTAAAATAAAAAGTTTAAATTAAATCC
ABE-F	AGGCAAAAATGTCAGAAGTTGAATTTTCACATGAATATTGG
ABE-R	GAGTATTTCTTATCGCTTCCTCCAGATGAGCCTC

dCas9RY-F	TCATCTGGAGGAAGCGATAAGAAATACTCAATAGGCTTAGCTA TCGG
dCas9RY-R	TTTAGCAAATCATGGTAGGTACCTAATGAAGCATTAA
sg-R	GCGGGCCCTTGACAGCTAGCTCAGTCCTAGGT
sg-F	GCCCTCAGCCGAAGATCTACCGCCTTTGAGTGAG
PCCI-F	TCGGTTGCGGCCGCTTGACAATTAATCAT
PCCI-R	AAAACTCGAGCAAAAAACCCCTCAAGACCCG
P-ABE-F	TCACACAGGAAACAGCATATGTCAGAAGTTGAATTTTCACATG AATATTGGA
P-ABE-R	GTACTTCTTGTCCATTGCTTCCTCCAGATGAGCCTC
sgRNA-F	TAGTATTAATACGACTCACTATAG
sgRNA-R	AAACCTATAGTGAGTCGTATTAAT
AMP-R	ATAATACCGCGCCACATAGC
eGFP-A	GGACTAGTTTACTTGTACAGCTCGTCCATGC
eGFP-S	GGGGTACCATGGTGAGCAAGGGCGA
sgRNA2-F	TAGTAGTGAGTCGTATTAATTTCG
sgRNA2-R	AAACCGAAATTAATACGACTCACT
A-F	CTAGACGTACGTACGTACGTACGT
B-R	GGACGTACGTACGTACGTACGT
gdhA-R	CCGAGGAATTTGAGAATGGAAAGG
argJ-R	CGCATCTTTTTCGCCCTGTA
argCB-R	AAGGTGGCGTCGTTAACA
argD-R	TGCCGGAGTTCATAAACACCA

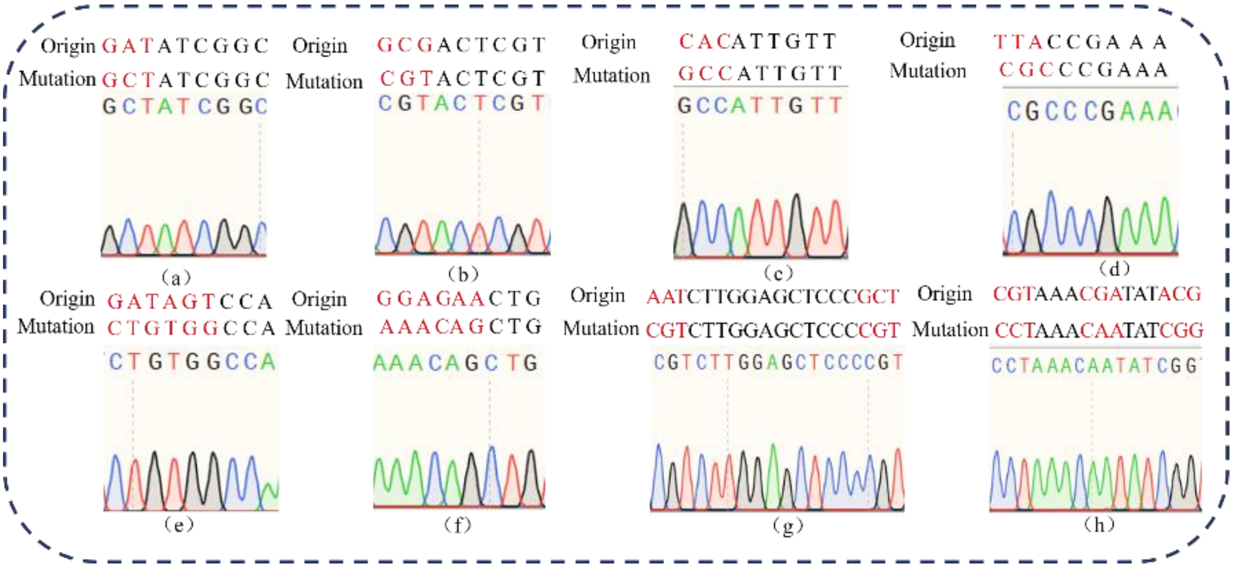

Supplementary Figure S1. Sequencing chromatograms of the mutation sites.
(a) D10A; (b) A61R; (c) H840A; (d) L1111R; (e) D1135L and S1136W; (f) G1218K and E1219Q; (g) N1317R and A1322R; (h) R1333P, R1335Q and T1337R
